# A survey on the role of artificial intelligence in managing Long COVID

**DOI:** 10.3389/frai.2023.1292466

**Published:** 2024-01-11

**Authors:** Ijaz Ahmad, Alessia Amelio, Arcangelo Merla, Francesca Scozzari

**Affiliations:** ^1^Department of Human, Legal and Economic Sciences, Telematic University “Leonardo da Vinci”, Chieti, Italy; ^2^Department of Engineering and Geology, University “G. d'Annunzio” Chieti-Pescara, Pescara, Italy; ^3^Laboratory of Computational Logic and Artificial Intelligence, Department of Economic Studies, University “G. d'Annunzio” Chieti-Pescara, Pescara, Italy

**Keywords:** artificial intelligence, deep learning, machine learning, Long COVID, post-acute sequelae of SARS CoV-2 infection, PASC

## Abstract

In the last years, several techniques of artificial intelligence have been applied to data from COVID-19. In addition to the symptoms related to COVID-19, many individuals with SARS-CoV-2 infection have described various long-lasting symptoms, now termed Long COVID. In this context, artificial intelligence techniques have been utilized to analyze data from Long COVID patients in order to assist doctors and alleviate the considerable strain on care and rehabilitation facilities. In this paper, we explore the impact of the machine learning methodologies that have been applied to analyze the many aspects of Long COVID syndrome, from clinical presentation through diagnosis. We also include the text mining techniques used to extract insights and trends from large amounts of text data related to Long COVID. Finally, we critically compare the various approaches and outline the work that has to be done to create a robust artificial intelligence approach for efficient diagnosis and treatment of Long COVID.

## 1 Introduction

Patients that have been infected with the SARS-CoV-2 virus can experience persistent and long-term effects known as Long COVID (Callard and Perego, 2021; Cau et al., 2022). Long COVID is known by several terms, such as post-COVID conditions, long-haul COVID, post-acute COVID-19, and the prolonged effects of COVID (Fernández-de Las-Peñas et al., 2021). Moreover, post-acute sequelae of SARS CoV-2 infection (PASC) (Pfaff et al., 2022) is also adopted as an alternative term for Long COVID.

**Figure 1:**
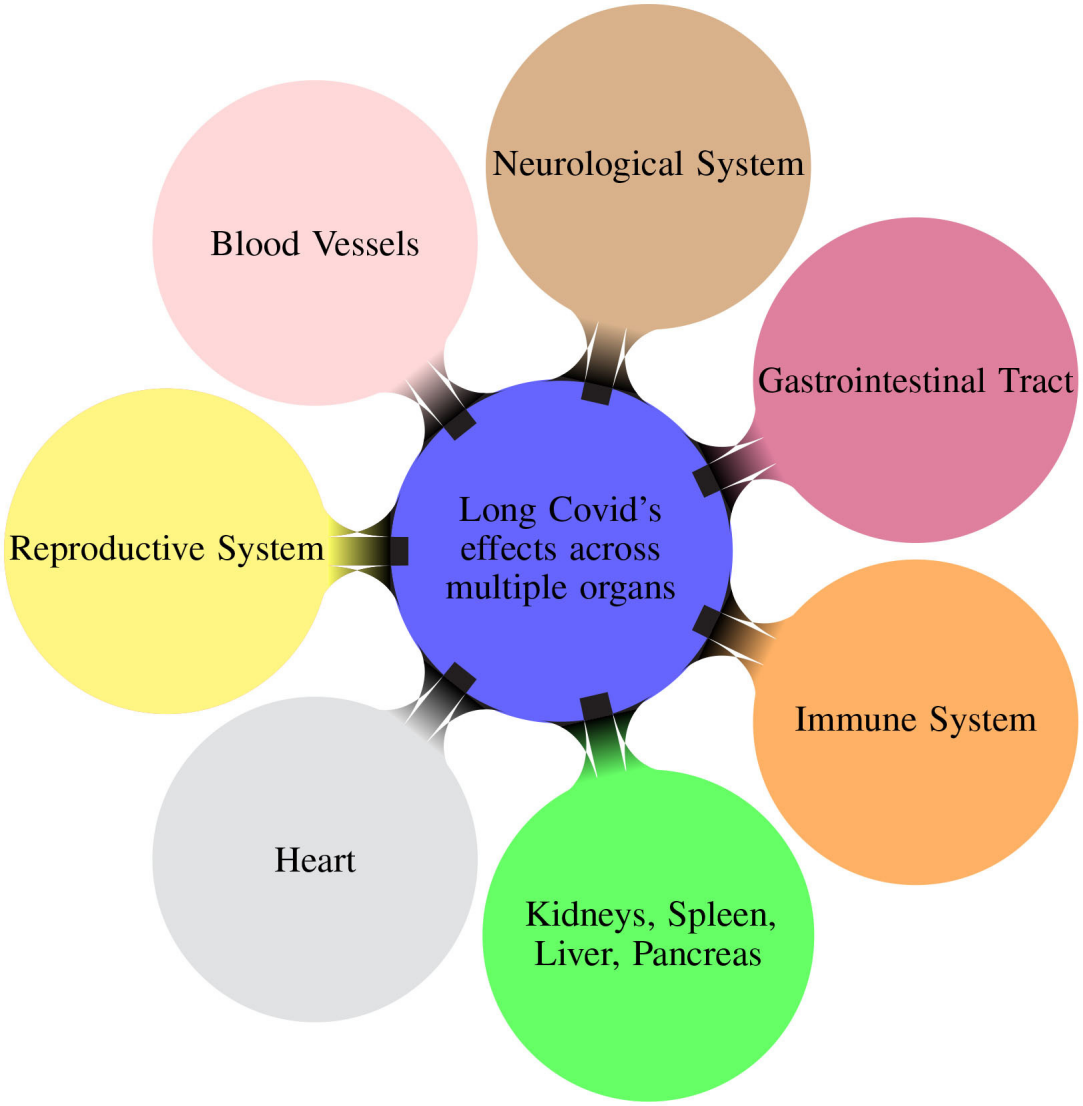
Mindmap illustrating the different organ systems on which the Long COVID has effects (Davis et al., 2023).

Patients experiencing Long COVID reported multiple post-COVID symptoms affecting different organs/systems (Davis et al., 2023). Figure 1 illustrates the multiple organs on which the Long COVID has effects. The virus can also have adverse effects causing sections of the immune system to become overactive and causing damaging inflammation throughout the body (Marshall, 2020).

The variety of potential symptoms and problems encountered by patients with Long COVID highlights the need for a deeper knowledge of the condition's clinical course. The most frequently reported symptoms of Long COVID that affect different organs are described in Table 1.

**Table 1:** Overview of Long COVID's effects and diverse pathologies across multiple organs (Davis et al., 2023).

**Organ/system**	**Associated symptoms and pathologies**
Lungs	Cough, dyspnoea, abnormal gas exchange
Heart	Chest pain, palpitations, cardiac impairment, myocardial inflammation, POTS
Kidneys, spleen, liver, pancreas	Organ injury
Immune system	Autoimmunity, MCAS
Gastrointestinal tract	Abdominal pain, nausea, gut dysbiosis, viral persistence and viral reservoir
Neurological system	Cognitive impairment, fatigue, disordered sleep, memory loss, tinnitus, dysautonomia, ME/CFS, neuroinflammation, reduced cerebral blood flow, small fiber neuropathy
Blood vessels	Fatigue, coagulopathy, deep vein thrombosis, endothelial dysfunction, microangiopathy, microclots, pulmonary embolism, stroke
Reproductive system	Erectile dysfunction, more severe and frequent premenstrual symptoms, irregular menstruation, reduced sperm count

The study of the prevalence, duration, and clinical outcomes of Long COVID is still under investigation (Walia et al., 2021). The scope and complexity of healthcare data require advanced analytics to derive meaningful insights from longitudinal data encompassing symptoms, laboratory results, imaging, functional assessments, genomic information, data from wearable sensors, mobile health applications, clinicians' notes, and electronic health records (EHR). Artificial intelligence (AI) and machine learning (ML) techniques increasingly show the potential to bring insight into patient-level data from massive amounts of data to comprehend the effect of SARS-CoV-2 on patients. Techniques of AI have been largely exploited for analyzing COVID-19 data [see for instance (Nayak et al., 2021)] but only a few works explore the trend patterns for Long COVID.

There is also an immediate need for enhanced care techniques that are more integrated to improve patient's clinical outcomes. These approaches would support and treat patients who have Long COVID establishing resilient healthcare systems to deliver efficient and effective responses to upcoming health challenges (Aiyegbusi et al., 2021). The deployment of AI can significantly improve everyday clinical practice (Recht et al., 2020) by answering physician inquiries concerning risk classification and the clinical outcome of COVID-19 patients. In fact, clinicians are faced with limited options as the existing diagnostic tools and therapeutics for Long COVID are still in the experimental stage, while early diagnosis and treatment would be crucial for improving patient outcomes. In this context, AI approaches would be helpful to automatize complex tasks that could hardly be produced manually.

In this survey, we consider data coming from symptoms, laboratory data and data from EHR on Long COVID. We specifically collect and analyze papers using AI techniques applied to Long COVID.

In the first part of the survey, we analyze ML techniques, such as Extreme Gradient Boosting (XGBoost), random forest, and Convolutional Neural Network (CNN), for predicting the prevalence of Long COVID and identifying the associated risk factors. Most of the papers in the literature perform a binary classification, and only a few works deal with predicting risk factors using regression methods, identifying blood proteins for Long COVID detection, and deriving Long COVID subphenotypes. The datasets employed comprise COVID-19 datasets, such as the National COVID Cohort Collaborative's (N3C) repository (Haendel et al., 2021), collections of surveys and health administrative data, laboratory data and patients' demographics comorbidities.

In the second part of the survey, we analyze natural language processing (NLP) approaches on textual data discussing Long COVID. In most cases, data are collected from Twitter, blogs, and clinical notes. Adopted approaches are mainly based on BERT models, topic modeling techniques, and association rule mining. The aim in these cases is to identify Long COVID symptoms and their co-occurrences.

In both cases, we focus only on papers that utilized AI techniques, including ML and NLP, to elaborate on the Long COVID data, discharging the considerable amount of work where basic statistics or other different models are exploited.

From the task point of view, we report and analyze different systems with the aim to: (i) identify and predict Long COVID from patients diagnosed as COVID-19 positive, (ii) predict the risk of developing different pathologies for patients who manifested COVID-19, and the potential long-term consequences of the emergence of the coronavirus, (iii) determine the associations between risk factors and Long COVID, (iv) distinguish between short and long COVID-19, (v) explore the characteristics, patterns and behavior of Long COVID symptoms, (vi) study the Long COVID course of the disease and evolution over time, and (vii) identify Long COVID symptom co-occurrences, topics of discussion about Long COVID, patient profiles and the challenges faced during treatment.

We gathered a total of 20 papers from the literature, with 13 of them focusing on ML techniques and the remaining seven on text mining applied to data related to Long COVID. We describe the individual contribution of each paper, the data and techniques adopted, and the results obtained, mainly in terms of accuracy, precision, recall, F1-score and AUC (Area Under the ROC Curve).

We critically analyze the different approaches and the results obtained, both in the ML and NLP categories, and also compare the two categories in terms of used datasets, methodologies, and obtained results. We show the current limitations of the approaches in the literature and outline future work directions in terms of AI methodologies and Long COVID target.

To the extent of our understanding, this is the first survey reviewing AI methodologies applied to Long COVID data.

## 2 The complexity of the Long COVID condition

Managing Long COVID is a complex issue, and the lack of effective pharmacological therapies and data to advise healthcare practitioners reflects this task's difficulty.

Long COVID has many different complications concerning manifestations, duration, and treatment. Diagnosing Long COVID can be difficult because of its wide variety of symptoms and its comorbidity with other illnesses. The development of precise diagnostic criteria is still ongoing. Establishing a worldwide standard for defining post-COVID-19 conditions is poised to enhance advocacy and research efforts significantly. However, this definition will likely undergo modifications in response to emerging evidence and the evolution of our comprehension of COVID-19's long-term effects. The Long COVID clinical definition was painstakingly crafted utilizing the exhaustive Delphi consensus approach. This approach relied on selecting relevant domains and variables for inclusion, as reflected in the WHO's ICD-10 diagnosis code U09. The process ensured the involvement and input of diverse stakeholders to ensure a well-rounded and inclusive understanding (Soriano et al., 2022). Long COVID solidified through patient-led surveys, self-appellation, case studies, and hashtag circulation. After patients, several new players and some typical scientific actors appear (Callard and Perego, 2021). Between December 2019 and May 2020, Davis et al. (2021) surveyed patients via an online questionnaire about their experiences with Long COVID symptoms, focusing on recovery and return to baseline from neurological and neuropsychiatric symptoms, including work impact.

Long COVID complexity and ongoing efforts to gather and prepare data make it an essential ground for multimodal ML techniques. Combining clinical and EHR data in Long COVID such as the National Institutes of Health (NIH) research initiative, MIDRC-N3C interoperability, pathology, wearable sensor data, imaging, and ML can help understand underlying physiology, explain heterogeneity, and identify therapeutic targets (Chen et al., 2023). The multimodal ML approaches' clinical usefulness depends on targeting the right clinical question, particularly Long COVID development, shared pathways, and response to treatment approaches.

## 3 Literature review

To discover relevant publications, we collected studies and data from different sources (PubMed, Scopus, WoS, MedRxiv, ArXiv). Identifying relevant papers was not a trivial task (Lever and Altman, 2021; Langnickel et al., 2022; Leaman et al., 2023).

In particular, we have considered, besides the basic term “Long COVID”, also multiple synonyms: “Post-COVID conditions”, “long-haul COVID”, “post-acute COVID”, “long-term effects of COVID”, “chronic COVID”, and also “post-acute sequelae of SARS CoV-2 infection” and “PASC” which refer to a subset of Long COVID cases. These terms have been used in combination with “artificial intelligence”, “machine learning”, “deep learning”, “natural language processing”, “NLP”, and “text mining”.

The search yielded substantial literature (121 papers selected from PubMed, Scopus, WoS, including only two papers from MedRxiv), including research articles, review articles, case studies, and reports. Among these articles, we selected all the contents pertaining to the application of AI methods to Long COVID data, and ended up with the 20 papers. The criteria used to select the papers were:

- relevance of the topic: we selected only papers with an innovative approach in the realm of AI. Accordingly, we discharged papers with basic statistic analysis;
- completeness and significance of the results: we selected the papers where AI is used to achieve some important result, removing those papers where AI was only discussed and not a clear result was obtained;
- publication date: we discharged all the papers published before 2020.

**Figure 2:**
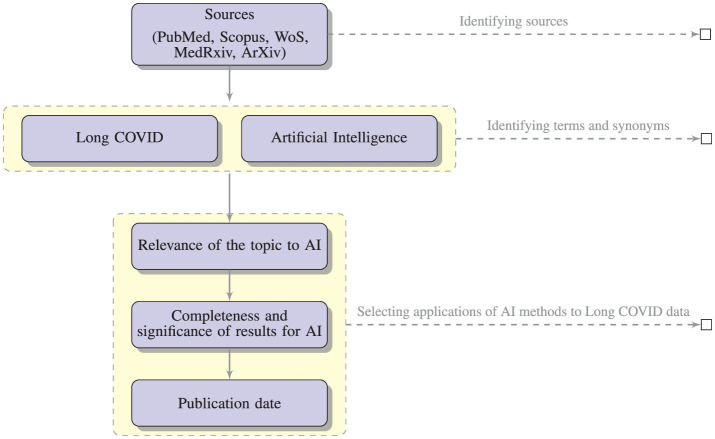
The literature selection process.

Figure 2 illustrates the process of selection of the relevant papers.

In the rest of the paper, we first analyze in Section 3.1 the literature where machine learning and deep learning models are developed, and then in Section 3.2 the studies where NLP techniques are applied. It is worth noting that medical images, electronic health records and other laboratory data can be the input to ML models for predicting a possible diagnosis of Long COVID, while clinical notes and tweets can be the input to NLP models to perform a risk factor or symptom co-occurrence analysis.

### 3.1 Machine learning and deep learning approaches to Long COVID data

This section presents recent research studies utilizing traditional and novel AI methods to detect Long COVID. We start with the authors applying ensemble learning techniques and then explore other approaches.

#### 3.1.1 Ensemble learning

In the ensemble learning context, a strategy known as Optimized XGBoost was suggested by Jha et al. (2023). This supervised learning strategy used an ensemble approach based on the gradient boosting method, and its customized hyper-parameters were used to increase the performance of Long COVID prediction. The researchers looked at COVID-19 patients who had lung fibrosis 90 days after being discharged from the hospital. Analyses were conducted on a dataset of 1175 EHRs and associated High-Resolution Computed Tomography (HRCT) chest images from COVID-19 patients. The dataset included 725 cases of pulmonary fibrosis and 450 cases of standard lung. The dataset was divided into distinct groups for training and testing purposes, with 881 samples allocated for training and 294 for testing. The findings of the experiments had an accuracy of 99.37% on the EHR dataset and 98.48% on the HRCT scan dataset, respectively. In order to reduce the dependence of the performance results on the size of the considered dataset, the authors divided the dataset into distinct sets of train-test data from which they derived the performance metrics. The suggested ML model optimized XGBoost, compared to other ML approaches, such as decision tree, Support Vector Machine (SVM), random forest, logistic regression, Naive Bayes, and the traditional XGBoost approach. The suggested system's precision, recall, and accuracy were higher than those of other approaches in the literature.

XGBoost machine learning models were also exploited by Pfaff et al. (2022) to identify patients affected by Long COVID using the N3C EHR database (Haendel et al., 2021). The dataset comprises information from more than 8 million patients with diverse demographics and geographic locations, obtained from their EHRs. The population (n=1,793,604) was selected among a set of alive adult patients over the age of 18 who had either an International Classification of Diseases-10 Clinical Modification or a positive SARS-CoV-2 PCR or antigen test or a COVID-19 diagnostic code (U07.1) from an inpatient or emergency visit, whose COVID-19 index date has passed at least 90 days. The authors investigated 97,995 persons diagnosed with COVID-19 regarding their demographics, healthcare usage, diagnosis, and medicines. In the study, researchers collected 924 features from 597 patients diagnosed with Long COVID. These features were used to train three ML models to determine the possible cases of Long COVID among COVID-19-diagnosed patients, COVID-19-hospitalized patients, and COVID-19-positive patients who were not hospitalized. Essential characteristics include the healthcare usage, the patient's age, dyspnea, and other information on diagnoses and medications that are available inside the EHR. The dataset was split into different sets for training (80% of hospitalized and 75% of not hospitalized patients) and testing (20% of hospitalized and 25% of not hospitalized patients). After additional validation of the models using data from a fourth location, the authors achieved an AUC value of 0.92 for all patients, 0.90 for hospitalized patients, and 0.85 for outpatients.

A different approach was presented by Gupta et al. (2022) for the early diagnosis of cardiac problems in COVID-19 survivors to predict Long COVID. In this work, an ensemble was performed using a stacked approach. The proposed model was trained on heart-related data acquired from 180 COVID-19 patients with a questionnaire. The data of the 180 patients were first bootstrapped to 4700 records, using a tenfold cross-validation approach. Data were divided into a training set (70%), a validation set (20%) and a test set (10%). The performance of the suggested model was compared to that of standard ML techniques. Performance measurements included accuracy, specificity, precision, and recall with two other statistical measures: Mean Absolute Error (MAE), and Root Mean Square Error (RMSE). Accuracy in predicting heart disease using the stacking ensemble method was 93.23%. The suggested method outperforms conventional learning algorithms, including decision trees, random forests, SVM, and artificial neural networks. The minimal RMSE (0.32) and MAE (0.23) values further support the suggested model's robustness.

A recent study by Jiang et al. (2022) focused on the relationship between vital signs (oxygen levels, heartbeat, systolic/diastolic blood pressure) and Long COVID. Since no vital measurement data are available for all the patients in the N3C cohort, two subcohorts with abundant vital measurement data for the first week after hospitalization were selected. Various features (139) were designed from vital measurement readings, including daily averages and daily variability features. Using data from the first subcohort, an XGBoost model predicted a Long COVID outcome, while CNN and LSTM were used to process a multidimensional time series of vital measures in the second subcohort. The authors evaluated the performance of the models using the standard AUC metric with 5-fold cross-validation.

A retrospective case-control research was designed by Hill et al. (2022) to determine risk factors linked to PASC and Long COVID from thirty-one health systems in the United States (N3C). COVID-19 risk factors included patient age, gender, comorbidities, medications, and acute symptoms. 8,325 persons were diagnosed with PASC compared to 41,625 healthy individuals from the same health system. Using multivariate logistic regression, random forest, and XGBoost, the correlations between potential risks and PASC were examined. This study identified a number of significant risk variables for PASC, including middle age, severe COVID-19 illness, and particular comorbidities. Results from the XGBoost and logistic regression models were comparable, with an AUC of 0.73. The random forest model, which has an AUC of 0.69, comes next.

A supervised ML algorithm based on random forest with five folds and ten iterations of stratified repeated cross-validation techniques was developed by Sudre et al. (2021) to determine who is susceptible to Long COVID and organize therapy and rehabilitation. This study used data from mobile health apps, allowing users to self-report their symptoms, with a sample size of 2,149. A simple model to differentiate short (duration of symptoms less than ten days) and Long COVID at seven days has an AUC of 75.9%.

Patel et al. (2023) also used a random forest classifier to classify the most pertinent blood proteins for the identification of Long COVID cases. The study compared the expression of 2,925 different blood proteins in Long COVID outpatients to COVID-19 inpatients and healthy individuals. The data were stratified by subject group and divided using a dimensionality reduction with 70% designated for training and 30% reserved for testing. The Boruta method was used for the feature reduction dataset to select the most important characteristics. A 3-fold cross-validation with a random forest of 10 trees and a maximum depth of 3 was adopted to limit the overfitting. Experts explicitly obtained unstructured text on mRNA or protein expression at the cell or tissue level, which NLP then processed to produce protein expression tissue specificity. The results revealed 119 essential proteins for classifying Long COVID outpatients, with classification accuracy of 100%, AUC 100% and F1-score 100%. Also, NLP expression analysis confirmed widespread organ system involvement and identified key cell types as crucial elements related to Long COVID.

Finally, Patterson et al. (2021) used the random forest for the classification of healthy, mild-moderate, severe, and Long COVID patients from their immunological profile. Data from 224 individuals were compiled, including 29 healthy individuals, 26 with mild to moderate COVID-19, 48 with severe COVID-19, and 121 with Long COVID. The dataset comprised 16 columns, with 14 dedicated to cytokine/chemokine levels, one for patient IDs, and one for classification (healthy, mild-moderate, severe, or Long COVID). Training, validation, and testing used 60%, 20%, and 20% of the data. The Synthetic Minority Oversampling Technique (SMOTE) was employed to balance class representation. Three random forest classifiers were then developed: a multi-class predictor, a binary classifier for severe COVID-19, and another binary classifier for Long COVID. These models were evaluated to identify critical cytokines significant in disease assessment. The multi-class model achieved an 80% accuracy and a 63% F1-score, while the Long COVID model reached a 96% accuracy with a 95% F1-score, and lastly, the severe model secured a 95% accuracy and a 94% F1-score.

#### 3.1.2 Deep learning

Using deep learning BiLSTM with a 1D CNN model, Sengupta et al. (2022) analyzed historical diagnosis code data from the N3C repository to identify possible risk factors of Long COVID. The study assessed patients for Long COVID infection using a chronological list of diagnosis codes up to 45 days following the initial positive test. The authors used Gradient-weighted Class Activation Mapping (Grad-CAM) to rate each input diagnosis. The diagnostic with the highest score was regarded as the most significant for making the proper diagnosis for a patient. The article proposed a method for collecting these leading diagnoses for each patient in the dataset and analysing their temporal trends to identify which codes are connected with a Long COVID positive diagnosis. Data were divided into training (75%), validation (15%), and testing (10%) sets. The study offered the mean AUC value of 3-fold stratified cross-validation for all models, achieving an accuracy of 70.48% despite the unbalanced dataset. Differently from the previous work where an LSTM was used, Subramanian et al. (2022) carried out diagnostic work for classification utilizing two CNN models, specifically VGG16 (Liu and Deng, 2015) and ResNet-50 (He et al., 2016), trained on 925 HRCT images, each with two different learning rates. The dataset was split into training (585 images), validation (65 images) and testing (275 images) sets. The best model produces an accuracy of 97.132%. An additional model was developed using a revised loss function that combines dice loss and binary cross-entropy, achieving an accuracy of 98.2%. The authors finally proposed a diagnostic model using the U-Net, which segmented and predicted the precise lung area infected with COVID-19 with an accuracy of 99.40%.

#### 3.1.3 Regression models

Binka et al. (2022) proposed a machine learning technique which uses the elastic net regression model to identify Long COVID cases in a population-based cohort of COVID-19 that have been reported in British Columbia, Canada. The suggested model was trained using the known Long COVID cohort patients' characteristics, including their demographics, existing medical problems, and other unique symptoms and complaints from health administrative data recorded after the index date for COVID-19 with 10-fold cross-validation. The optimal model exhibited a high sensitivity and specificity rate of 86% and AUC of 93%, classifying 25,220 individuals out of 141,381 COVID-19 patients as Long COVID cases.

By contrast, Moreno-Pérez et al. (2021) used a traditional multiple logistic regression model to assess the acute infection phase risk variables linked to Long COVID. Data were collected from electronic medical records, and a follow-up assessment was conducted 10-14 weeks after either recovery from COVID-19 in an ambulatory setting or hospital discharge. This assessment comprised a clinical examination, blood tests, chest X-ray, pulmonary function tests, and a quality of life questionnaire. The study results showed that Long COVID was detected in half of COVID-19 survivors. Mild radiological and spirometric alterations were detected in less than 25% of the patients. Independent predictors were not found among the baseline clinical characteristics for the Long COVID development. The predictors of the outcome were examined using multiple logistic regression with a 95% cumulative incidence value.

Table 2 summarizes all the approaches presented in the previous sections. Note that several papers use the same datasets, looking at different features and using different techniques. Moreover, given the class imbalance of many of the datasets related to Long COVID, it is important to note that the accuracy measure can provide an inaccurate impression of the quality of a model and in general, of the overall analysis results.

**Table 2:** ML techniques for Long COVID diagnosis.

**Study**	**Input data**	**AI method**	**Task**	**Output (%)**
Jha et al. (2023)	1,175 EHR & HRCT	Optimized XGBoost	Binary classificationof pulmonary fibrosis	Accuracy 99.37precision 99.54
Pfaff et al. (2022)	N3C repository	XGBoost	Binary classificationof Long COVID	AUC all patients 92hospitalized 90non-hospitalized 85
Jiang et al. (2022)	N3C repository	XGBoostCNN LSTM	Binary classificationof Long COVID	AUCXGBoost 82.2CNN 61.64 LSTM 59.94
Hill et al. (2022)	N3C repository	XGBoostRandom forest	Risk factors associatedwith Long COVID	AUC XGBoost 73 Random forest 69
Gupta et al. (2022)	180 questionnaires	Stacking ensemble technique	Binary classificationof heart diseases	Accuracy 93.23 precision 95.248
Sudre et al. (2021)	2,149 self-reported health status and symptoms	Random forest	Binary classification ofshort and Long COVID	AUC 75.9
Patel et al. (2023)	Expression of 2,925 unique blood proteins	Random forestNLP	Identification of blood proteins for Long COVID detection	AUC 100accuracy 100F1-score 100
Patterson et al. (2021)	Immunologic profiles from 224 individuals	Random forest	Classification of healthy, mild-moderate, severe and Long COVID patients	Multi-class:accuracy 80F1-score 63Long COVID:accuracy 96F1-score 95Severe: accuracy 95F1-score 94
Sengupta et al. (2022)	N3C repository	BiLSTM with 1D CNN model	Binary classificationof Long COVID	Accuracy 70.48
Subramanian et al. (2022)	925 HRCT	VGG-16 ResNet-50 U-Net	Binary classificationof Long COVID	Accuracy from 97.132 to 99.4
Binka et al. (2022)	26,730 health administrative data	Elastic Netregression	Binary classification of Long COVID	AUC 93sensitivity 86 specificity 86
Moreno-Pérez et al. (2021)	277 patients'demographics and comorbidities	Multiple logisticregression	Risk factors associatedwith Long COVID	Cumulative Incidence Value 95
Zhang et al. (2023)	34,605 EHR	Topic modelingclustering	Derive Long COVID subphenotypes	Four Long COVID subphenotypes

The first part of the table (6 rows) refers to ensemble learning, the second part (2 rows) to deep learning, and the last parts (2 rows and 1 row) refer to regression models and other approaches, respectively (all reported measures have the same number of decimal digits as the original paper).

#### 3.1.4 Other approaches

Zhang et al. (2023) proposed a machine learning-based approach on topic modeling to derive Long COVID subcategories based on newly acquired medical conditions during the post-acute phase of a COVID-19 infection. The study focused on 30-180 days after confirmed COVID-19 infection. Development and validation cohorts were formed using EHRs from two large cohorts, INSIGHT and OneFlorida+, part of the National Patient-Centered Clinical Research Network, including 20,881 and 13,724 patients infected by COVID-19. The ML method analyzed more than 137 symptoms and conditions in the cohort of patients with newly incident conditions within 30-180 days after COVID-19 infection. After computing a 137-dimensional binary vector encoding of each patient with Long COVID diagnoses, it learned Long COVID topics from these vectors. Specifically, Long COVID subjects are sets of circumstances that occur together according to their respective event probabilities. Next, a topic modeling technique is used to infer patient representations in the low-dimensional Long COVID topic space. Based on how extensively each topic was covered in the patients' post-acute phase data, these themes are used to further characterize the patients. Finally, a clustering method was employed from the patient representations to detect the subphenotypes. The analysis detected four Long COVID subphenotypes: (i) cardiac and renal sequelae affected 33.75% of patients in the development cohort and 25.43% in the validation cohort; (ii) respiratory, sleep, and anxiety issues were observed in 32.75% and 38.48% of these cohorts, respectively; (iii) musculoskeletal and nervous system complications occurred in 23.37% and 23.35%; and (iv) digestive and respiratory system issues were seen in 10.14% and 12.74% of patients, each linked to specific patient demographics.

### 3.2 Text mining's role in Long COVID diagnosis and therapy

The latest developments in NLP offer the possibility of improving healthcare and public health. Massive amounts of unstructured data are continuously generated from various sources, including EHRs, social media, and recent literature. One of the goals of this investigation is to look ahead to potential uses of NLP-based technologies which can help enhance pandemic response preparedness, extracting textual patterns which can represent Long COVID symptoms and relationships between symptoms, and the discussion topics about COVID-19. *Pandemic response preparedness* means not just handling immediate issues but also planning for long-term effects, like Long COVID.

The aim is to improve public awareness of Long COVID, provide important insights to public health authorities, and learn more about the health effects of Long COVID.

In the following, we distinguish among the approaches based on BERT (Devlin et al., 2018) and other techniques.

#### 3.2.1 BERT approaches

Miao et al. (2022) analyzed 30,327 user-generated conversations on Twitter about Long COVID symptoms. NLP was utilized to investigate how Twitter users described the nature of Long COVID symptoms in terms of demographic features such as patient's gender, age, and geographical location, as well as temporal parameters such as symptom severity and duration. Moreover, to address the Long COVID evolution over time, the study compared the results of datasets collected in different periods. The authors constructed two sets of tweets related to Long COVID; the first set spanned from 1st May to 31st December 2020, and the second was from October 2021. They randomly divided the annotated data into 80% for training and 20% for testing purposes. To ensure the accuracy of the automated labeling, they manually checked a subset of the labeled samples. On the demographic categories, the BERT classifier reached an accuracy of 89%, while on the symptom categories, it reached an accuracy of 95%.

BERT was also used by Zhu et al. (2022) on free-text clinical notes to identify patients with persistent symptoms following acute COVID-19 infection. Data from clinical notes of 719 patients seen by physicians were analyzed to look for patient similarities. The authors employed 5-fold cross-validation and divided the training, validation, and testing data by a ratio of 60%:20%:20%. The study applied three different pre-trained BERT models to automatically identify patients with Long COVID effects. The ClinicalBERT model achieved a sensitivity score of 0.88 for note-level prediction. The study identified potential phenotypes from the classification results.

To gain insight into the Italian perspective of the COVID-19 pandemic, Scarpino et al. (2022) discussed and compared two topic modeling techniques: Latent Dirichlet Allocation (LDA) and a BERT transformer (BERTopic). The authors analyzed texts written by patients with Long COVID, healthcare professionals, and citizens (without Long COVID), with the aim of characterizing patients affected by Long COVID based on the textual narration. BERTopic is a topic modeling technique that adopts transformers and c-TF-IDF to create clusters to represent topics and identify important words in the topic descriptions. The BERTopic-based method surpassed the LDA-based method, with 97.26% of documents correctly clustered and an overall accuracy of 91.97%.

#### 3.2.2 Other approaches

Differently from the previous approaches where classification is adopted, Matharaarachchi et al. (2022) explored the trends and characteristics associated with Long COVID using the Apriori algorithm-based Association Rule Mining Technique. The focus of the study was to examine the common symptoms of patients with Long COVID and determine any correlations between them, using Twitter social media conversations as a reference. The authors set a minimum support threshold value of 0.001, a lift greater than 1, and a confidence level of 10% for positively correlated rules. According to the results, the three indications and symptoms that occurred most frequently were brain fog, fatigue, and breathing or lung issues.

Using EHR data, a comprehensive Long COVID symptom lexicon was developed by Wang et al. (2022). The authors evaluated PASCLex, a lexicon-based NLP approach that uses data-driven approaches based on medical ontologies to extract Long COVID symptoms from clinical notes. The primary dataset consisted of 23,505 patients, accounting for 90%, with 299,140 related clinical notes. In contrast, the validation subset comprised 2,612 patients, which is 10% of the total, and included 29,739 respective clinical notes. The developed method took advantage of the Unified Medical Language System (UMLS) and achieved precision and recall values of 94% and 84%, respectively.

Investigating the progression of the disease in its post-acute phase by analyzing 296,154 tweets, Banda et al. (2021) employed a blend of machine learning and NLP techniques, supplemented by clinician evaluations, to construct comprehensive symptom and condition timelines spanning 150 days. This process involved expert annotation of tweets, machine learning for filtering relevant content, and NLP for standardizing the data. The primary outcome of this approach was the evaluation of temporal symptoms, timeline visualization, and cluster identification.

Similarly, Déguilhem et al. (2022) collected and analyzed data from France on Long COVID most frequently reported symptoms, symptom combinations, challenges, and patient profiles. Data were gathered from the social media Twitter and the health-related online forum Doctissimo (https://www.doctissimo.fr). Symptoms were indexed using the MedDRA dictionary, ranked according to the times they were mentioned in posts, and summarized on a per-user basis. The study proposed to compute co-occurrences of terms in users' posts. The posted content was analyzed to identify common terms, and users were then grouped using hierarchical clustering based on these terms. The study looked at 289 users who used at least two distinct symptom phrases in their messages. A heat map was produced to illustrate the major co-occurrences. NLP-based text mining approach Biterm Topic Modeling (BTM) was used to analyze the conversations, and difficulties and unfilled needs were discovered through in-depth interviews. The analyses identified three major symptom clusters: asthenia-dyspnea (102/289, 35.3%), asthenia-anxiety (65/289, 22.5%), and asthenia-headaches (50/289, 17.3%).

Table 3 summarizes the aforementioned NLP approaches.

**Table 3:** Long COVID diagnosis: recently applied data mining and NLP techniques.

**Study**	**Input data**	**AI method**	**Task**	**Output (%)**
Miao et al. (2022)	Tweets	NLP	Analysis of reported Long COVID symptoms in terms of demographics, geographical and temporal parameters	Accuracy demographic categories 89 symptom categories 95
Zhu et al. (2022)	Clinical notes	Pretrained BERT	Identification of Long COVID and potential computational phenotypes	Sensitivity score 88.1
Scarpino et al. (2022)	Blogs	LDA and BERT	Extract discussion topics in the Italian narration of COVID-19 pandemic	Accuracy of BERT 91.97
Matharaarachchi et al. (2022)	Tweets	Association rule mining	Relationships between symptoms	Confidence 77 for lung/breathing problems and loss of taste vs. loss of smell
Wang et al. (2022)	Clinical notes	PASCLex (NLP) model	Identification of symptoms	Precision 94 recall 84
Banda et al. (2021)	Tweets	NLP and SVM	Identification of symptoms	Accuracy 75 on a 20% random held-out test set
Déguilhem et al. (2022)	Tweets	Biterm Topic Modeling	Identification and co-occurrence of symptoms	Three major symptom co-occurrences: asthenia-dyspnea 35.3, asthenia-anxiety 22.5, asthenia-headaches 17.3

The division is between BERT (the first 3 rows) and other approaches (all reported measures have the same number of decimal digits as the original paper).

**Figure 3:**
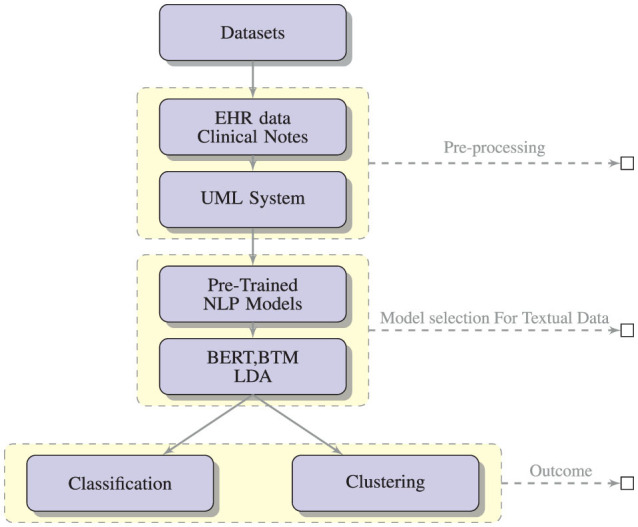
A conceptual framework for the generation and assessment of PASC symptoms using NLP-based text mining methods.

Figure 3 shows the expected outcomes of applying NLP-based text mining algorithms like BERT, BTM and LDA on data from clinical settings and social media data with the expected outcome of classification and clustering.

### 3.3 Task description

In this section, we recall the main tasks of the different papers. For the technical description of the input data and obtained results, the reader can refer to Tables 2, 3.

- Jha et al. (2023) identify the development risk of pulmonary fibrosis after 90 days of hospital discharge from clinical features retrieved at the time of follow-ups of COVID-19 patients.
- Pfaff et al. (2022) detect patients with Long COVID using EHR with diagnosis and medication characteristics from patients and for whom at least 90 days have passed since COVID-19 index date.
- Jiang et al. (2022) detect Long COVID from the features of vital measurements of patients with a diagnosis of COVID-19 and hospitalized. Measurements derived from the first seven days since the hospitalization started.
- Hill et al. (2022)'s objective was to identify links between risk factors such as demographics, comorbidities, and treatment, as well as acute characteristics associated with COVID-19, and Long COVID. Patients with Long COVID were included based on a prior diagnosis of SARS-CoV-2 infection or a positive polymerase chain reaction (PCR) or antigen (AG) lab test for SARS-CoV-2, with the initial acute infection date ranging from March 1, 2020, to December 1, 2020.
- Gupta et al. (2022) detect the risk of heart disease, as well as the long-term negative consequences of the coronavirus outbreak on recovered patients from data of patients who were diagnosed with COVID-19, in particular personal details, severity of disease, recovery days, hospital admission, symptoms during disease and Long COVID symptoms.
- Sudre et al. (2021) differentiate between short and long COVID-19 at seven days from fatigue, headache, dyspnea and anosmia symptoms. A connection was shown between more than five symptoms during the first week of sickness and the presence of Long COVID.
- Patel et al. (2023) find novel blood biomarkers for Long COVID by comparing protein expression in COVID-19 inpatients, healthy control individuals, and Long COVID outpatients. The discovered proteins represented a wide variety of cell types and organs.
- Patterson et al. (2021) identify and characterize the immunologic steps of COVID-19 (healthy, mild-moderate, severe and Long COVID) from the immunological profile in order to detect and monitor effective treatment plans. After the first symptoms appeared, the duration of Long COVID continued for more than 12 weeks.
- Sengupta et al. (2022) determine whether a patient is impacted by Long COVID by analysing a chronologically ordered set of diagnostic codes up to 45 days after the initial positive test or diagnosis. Looking at the overall temporal evolution for all patients allows to identify which codes lead to a Long COVID positive diagnosis.
- Subramanian et al. (2022) make a binary classification of COVID-19 images, detect lungs region on HRCT images and identify COVID-19 region on HRCT images from patients with various recovery periods from COVID-19 infection.
- Binka et al. (2022) detect Long COVID cases from health administrative data, including demographic features, pre-existing conditions, COVID-19-related data and all symptoms recorded 28 days after the COVID-19 symptom index date and lasted up to 183 days afterwards.
- Moreno-Pérez et al. (2021) study the incidence of Long COVID and its features and assess the risk factors connected to the acute infection step from data of adult patients who had recovered from COVID-19 (from 27th February to 29th April 2020), with a systematic evaluation 10-14 weeks after disease occurrence.
- Zhang et al. (2023) find Long COVID subphenotypes based on newly incident conditions in the post-acute COVID-19 infection period, defined as 30-180 days after the confirmed infection, of patients with COVID-19.
- Miao et al. (2022) analyze the features of Long COVID symptoms included in Long COVID-related tweets from May to December 2020 in terms of the patient's gender, age, location and duration of symptoms, and also analyze the Long COVID evolution over time, making a comparison of the results between May-December 2020 and October 2021.
- Zhu et al. (2022) detect Long COVID from clinical notes of outpatient encounters of patients with constant symptoms after their positive COVID-19 tests between 30 days after the positive COVID-19 diagnosis and 365 days after diagnosis and characterize potential phenotypes.
- Scarpino et al. (2022) characterize textual narration of Long COVID patients by discussed topics from textual testimonies written about COVID-19 illness, which are parts of texts written by subjects affected by Long COVID, and texts of healthcare professional and general reflections by citizens.
- Matharaarachchi et al. (2022) analyze the patterns and behavior of Long COVID symptoms reported by patients from Twitter data retrieved from May 2020 to December 2021. Obtained results proved that patients with lung/breathing problems and loss of taste are likely to lose smell with 77% confidence.
- Wang et al. (2022) generate a comprehensive Long COVID symptom lexicon (PASCLex) from clinical notes (day 51–110 from first positive COVID-19 test) to assist the identification of symptoms. Among the symptoms with the highest frequency, there are pain, anxiety, depression, fatigue, joint pain, shortness of breath, headache, nausea and/or vomiting, myalgia, and gastroesophageal reflux.
- Banda et al. (2021) employ social media data derived from Twitter to define the Long COVID course of the disease, generating detailed timelines of symptoms and conditions and studying their symptomatology for a period of over 150 days. They rebuild a timeline for each Twitter user with the main phases (testing, symptoms, therapy, etc.).
- Déguilhem et al. (2022) detect and study Long COVID symptoms, symptom co-occurrences, topics of discussion, difficulties encountered, and patient profiles. Data were extracted based on a collection of pertinent keywords from public sites (e.g., Twitter) and health-related forums (e.g., Doctissimo) between January 2020 and August 2021. The analyses found three major symptom co-occurrences: asthenia-dyspnea, asthenia-anxiety, and asthenia-headaches.

## 4 Discussion

It is worth noting that all the papers reported in Table 2 discuss ML systems, and all the papers reported in Table 3 discuss NLP systems.

The *Input data* column in Table 2 summarizes the different datasets used in the papers. The variety of the datasets allowed us to understand Long COVID from various perspectives. The N3C dataset (Haendel et al., 2021) has been the most used one. It is a large-scale collection of EHRs collected with a collaborative effort from different healthcare systems and research institutions in the USA. The network consists of a collaborative partnership involving over 600 individuals and 100 organizations. This coalition focuses on national collaboration and governance, formulating regulatory strategies, defining COVID-19 cohorts through community-developed phenotypes, and standardizing data. The N3C facilitates community-led, replicable, and clear analysis of COVID-19 data, promoting the swift sharing of findings and precise attribution. EHR data derived from 14,026,265 patients who: (i) have tested positive for COVID-19 infection (5,409,269 patients), (ii) have symptoms that are compliant with a COVID-19 diagnosis, or (iii) have tested negative for COVID-19 infection (and have never tested positive) to support comparative analysis. EHR data have many features, including demographics, geographical locations of patients, healthcare visits, medical conditions, vital measurements of patients, and prescriptions. The N3C repository also includes specific COVID-19 diagnoses and service utilization dates. Additionally, it contains records of patients identified with the newly implemented ICD-103 U09.9 code, which is used to mark patients diagnosed with Long COVID. We believe that the strength of this dataset is its large sample size, which comprises millions of patients. When used in machine learning and deep learning approaches, this allows for a more robust analysis across diverse patient populations, even from different geographic regions. The number of works exploiting this dataset confirms that it is one of the most valuable resources for researchers studying Long COVID.

Table 2 shows that most papers use ensemble techniques, which are able to produce more accurate results, compared to the approaches using CNN. We believe that the reason could be the robustness of this approach, which can better handle noise and outliers in the data. In fact, note that the datasets include collections from surveys and self-reported status data (using an app), where these phenomena may easily happen. Another reason could be that Long COVID is a complex and multifaceted condition with different manifestations and risk factors. Ensemble techniques can handle this complexity by combining models, each of which specializes in different aspects of the data so that the overall result enables a more comprehensive analysis and enhances the model's ability to capture the data complexities. In fact, the approach with the best performance is the random forest by Patel et al. (2023) followed by the Optimized XGBoost by Jha et al. (2023).

Table 2 also shows that most approaches focus on simple targets, such as binary classifications, whose primary goal is to diagnose and identify cases of Long COVID versus non-cases. This also includes the approach of Binka et al. (2022), which adopts an Elastic Net regression model but then produces a binary classification. Only Hill et al. (2022) (with XGBoost and random forest) and Moreno-Pérez et al. (2021) (with multiple logistic regression) examine the relationship between risk variables and the development of Long COVID. Finally, Patterson et al. (2021), Patel et al. (2023), and Zhang et al. (2023) adopted multiclass classification of Long COVID data.

Table 3 shows that the BERT model is the most used approach for pattern extraction from text data discussing Long COVID. In particular, it is adopted in Miao et al. (2022) on Twitter data for characterizing the nature of Long COVID symptoms in terms of demographic features, in Zhu et al. (2022) on free-text clinical notes to identify patients with persistent symptoms following acute COVID-19 infection, and in Scarpino et al. (2022) for characterizing patients affected by Long COVID based on the textual narration. Also, we can observe that BERT models are adopted for most of the different types of textual data, i.e., tweets (Miao et al., 2022), clinical notes (Zhu et al., 2022) and blogs (Scarpino et al., 2022). In terms of topic modeling, the approach introduced by Scarpino et al. (2022) using LDA and BERT models, and the approach introduced by Déguilhem et al. (2022) using Biterm Topic Modeling, adopted textual data based on patients' opinions, i.e., blogs and tweets.

Regarding the approaches using models different from BERT, they are mostly employed for identifying Long COVID symptoms [see Matharaarachchi et al. (2022) using Association Rule Mining, Wang et al. (2022) using PASCLex (NLP) model, and Banda et al. (2021) using NLP and SVM model] and for capturing symptom co-occurrences [see Déguilhem et al. (2022) using Biterm Topic Modeling].

In terms of data, it is worth noting that most of the techniques are employed on textual data from Twitter. Among these techniques, three out of four which are based on association rule mining (Matharaarachchi et al., 2022), NLP and SVM (Banda et al., 2021), and Biterm Topic Modeling (Déguilhem et al., 2022), are adopted for Long COVID symptom identification and co-occurrence, while one of them (Miao et al., 2022) aims to describe the nature of Long COVID symptoms in terms of demographic features.

From a comparison of Tables 2, 3, we can observe that most of the approaches in Table 2 are related to a binary classification of Long COVID, i.e., if the patient is affected or not by Long COVID. Also, only two papers in Table 2 (Moreno-Pérez et al., 2021; Hill et al., 2022) aim to predict the risk factors associated with Long COVID. Finally, Patterson et al. (2021), Patel et al. (2023), and Zhang et al. (2023) adopted multiclass classification of laboratory data for Long COVID identification. By contrast, the approaches based on NLP in Table 3 are mostly related to Long COVID symptoms identification [see Banda et al. (2021); Déguilhem et al. (2022); Matharaarachchi et al. (2022); Miao et al. (2022); Wang et al. (2022), and Zhu et al. (2022)].

Regarding the complexities of Long COVID, we can observe that Jiang et al. (2022), Gupta et al. (2022), and Miao et al. (2022) used input data characterized by a temporal duration of COVID-19, in particular measurements of patients from the first 7 days since the hospitalization start day, recovery days and symptom duration. It is worth noting that both Gupta et al. (2022) and Jiang et al. (2022) adopted ensemble learning (i.e. XGBoost and stacking ensemble technique), which is a more robust and reliable approach than traditional classifiers. Jiang et al. (2022) also used CNN and LSTM, which are deep learning methods specifically adopted for prediction tasks on temporal data. Still, Sengupta et al. (2022) used temporally ordered input data and looked at the temporal trends for all the patients, and Miao et al. (2022) studied the Long COVID evolution over time. For capturing the complexity of the task, Sengupta et al. (2022) used BiLSTM with a 1D CNN model, which is a powerful network for managing temporal data. Finally, Banda et al. (2021) analyzed the symptomatology of Long COVID conditions and symptoms over a period of more than 150 days using detailed timelines. From this analysis, we can observe that, although the length of symptoms is very important for Long COVID, many ML and NLP methods do not address this aspect. Only Miao et al. (2022) takes into account the symptom duration.

## 5 Recommendation and future work

In this survey, we have presented the applications of artificial intelligence in the Long COVID diagnostics, classification, risk factor prediction, and symptom occurrences. The ML approaches include (Optimized) XGboost, CNN, LSTM, random forest, stacking ensemble technique, Elastic Net regression, SVM, multiple logistic regression, topic modeling and clustering, BERT, and LDA.

Moreover, NLP approaches such as LDA and topic modeling based on the BERT transformer and Biterm Topic Modeling play a vibrant role in investigating how data from Twitter users and inpatients describe the nature of Long COVID symptoms in terms of demographic features (such as patient's gender, age, and geographical location), as well as temporal parameters such as symptom severity and duration. This will help to address the Long COVID evolution over time.

ML algorithms require massive datasets and high-quality information to construct effective models or discover meaningful patterns. The types of data used in the papers in Tables 2, 3 are EHR, health administrative data, patients' demographics and comorbidities, HRCT, other laboratory data, surveys, (free-text) clinical notes, tweets and blogs. These papers mainly focused on the following tasks: (i) identification and prediction of Long COVID from patients diagnosed as COVID-19 positive, (ii) prediction of the risk to develop different pathologies for patients who manifested COVID-19, along with the prolonged adverse impacts of the coronavirus pandemic, (iii) determining the associations between risk factors and Long COVID, (iv) distinction between short and long COVID-19, (v) exploring the characteristics, patterns and behavior of Long COVID symptoms, (vi) study of the Long COVID course of the disease and evolution over time, and (vii) identification of Long COVID symptom co-occurrences, topics of discussion about Long COVID, difficulties encountered, and patient profiles.

However, the methods described in this survey have some limitations from the point of view of input data and adopted AI models. The first problem is that data on Long COVID are still relatively scarce, and existing datasets may be biased or incomplete (Pfaff et al., 2022). For instance, the N3C repository data is limited and may include healthcare access limitations. As a result, it may be challenging to construct accurate and generalized models over a wide range of patient data. Also, identifying the many factors, among age, sex, or infection severity, predicting the likelihood of developing Long COVID symptoms and their co-occurrence is still an open problem. Another source of data that, to the best of our knowledge, has not been explored comes from wearable devices that can remotely acquire data and share it with medical teams. Advances in digital technology have made it easier to collect electronic patient-reported data like temperature, oxygen saturation, and blood pressure. We believe that utilizing such data with machine learning and artificial intelligence (Lassau et al., 2021) could improve identifying and monitoring individuals at risk to enable early clinical intervention and rehabilitation. Moreover, considering the current availability of data on the phenomenon, most approaches in Table 2 focus on binary classification to detect cases of Long COVID. We believe that multiclass approaches or regression analyses are also possible and could bring more insightful results.

In conclusion, the approaches in Table 3, aiming to detect symptoms from textual data describing Long COVID, are quite different from the approaches in Table 2, aiming to identify the presence of Long COVID from symptoms, laboratory data and demographic features. In some sense, the former could be used for feeding the latter. More specifically, symptoms detected from textual data by an NLP approach can be inputted into a model for identifying the presence of Long COVID from the given symptoms. Also, note that most of the works in Table 2 deal with the diagnosis of Long COVID, namely with identifying and confirming that an individual is experiencing persistent symptoms following a COVID-19 infection. We believe that a prediction of prognosis in Long COVID could be interesting for the expected course of the condition, for instance, the duration of the persistent symptoms and the likelihood of symptom resolution. For example, using temporal data such as a symptom or physiological monitoring data over time, deep learning analyses could detect early signs of worsening of Long COVID, allowing for timely interventions and enabling personalized adaptations of therapies to improve patient outcomes.

## Author contributions

IA: Conceptualization, Formal analysis, Methodology, Project administration, Supervision, Writing—original draft, Writing—review & editing. AA: Conceptualization, Formal analysis, Methodology, Project administration, Supervision, Writing—original draft, Writing—review & editing. AM: Conceptualization, Methodology, Supervision, Writing—original draft, Writing—review & editing. FS: Conceptualization, Formal analysis, Methodology, Project administration, Supervision, Writing—original draft, Writing—review & editing.
